# Coinfection with *Schistosoma mansoni* Enhances Disease Severity in Human African Trypanosomiasis

**DOI:** 10.1155/2023/1063169

**Published:** 2023-11-03

**Authors:** Nancy S. Mitalo, Naomi N. Waiganjo, John Mokua Mose, David O. Bosire, James O. Oula, Alfred Orina Isaac, James Nyabuga Nyariki

**Affiliations:** ^1^Department of Biomedical Science & Technology, Technical University of Kenya, P.O. Box 52428, Nairobi 00200, Kenya; ^2^Department of Biochemistry and Biotechnology, Technical University of Kenya, P.O. Box 52428, Nairobi 00200, Kenya; ^3^Department of Pharmaceutical Sciences and Technology, Technical University of Kenya, P.O. Box 52428, Nairobi 00200, Kenya

## Abstract

**Introduction:**

Human African trypanosomiasis (HAT) and schistosomiasis are neglected parasitic diseases found in the African continent. This study was conducted to determine how primary infection with *Schistosoma mansoni* affects HAT disease progression with a secondary infection with *Trypanosoma brucei rhodesiense* (*T.b.r*) in a mouse model.

**Methods:**

Female BALB-c mice (6–8 weeks old) were randomly divided into four groups of 12 mice each. The different groups were infected with *Schistosoma mansoni* (100 cercariae) and *Trypanosoma brucei rhodesiense* (5.0 × 104) separately or together. Twenty-one days after infection with *T.b.r*, mice were sacrificed and samples were collected for analysis.

**Results:**

The primary infection with *S. mansoni* significantly enhanced successive infection by the *T.b.r*; consequently, promoting HAT disease severity and curtailing host survival time. *T.b.r*-induced impairment of the neurological integrity and breach of the blood-brain barrier were markedly pronounced on coinfection with *S. mansoni.* Coinfection with *S. mansoni* and *T.b.r* resulted in microcytic hypochromic anemia characterized by the suppression of RBCs, hematocrit, hemoglobin, and red cell indices. Moreover, coinfection of the mice with the two parasites resulted in leukocytosis which was accompanied by the elevation of basophils, neutrophils, lymphocytes, monocytes, and eosinophils. More importantly, coinfection resulted in a significant elevation of alanine aminotransferase (ALT), aspartate aminotransferase (AST), alkaline phosphatase (ALP), total bilirubin, creatinine, urea, and uric acid, which are the markers of liver and kidney damage. Meanwhile, *S. mansoni*-driven dyslipidemia was significantly enhanced by the coinfection of mice with *T.b.r.* Moreover, coinfection with *S. mansoni* and *T.b.r* led to a strong immune response characterized by a significant increase in serum TNF-*α* and IFN-*γ*. *T.b.r* infection enhanced *S. mansoni*-induced depletion of cellular-reduced glutathione (GSH) in the brain and liver tissues, indicative of lethal oxidative damage. Similarly, coinfection resulted in a significant rise in nitric oxide (NO) and malondialdehyde (MDA) levels.

**Conclusion:**

Primary infection with *S. mansoni* exacerbates disease severity of secondary infection with *T.b.r* in a mouse model that is associated with harmful inflammatory response, oxidative stress, and organ injury.

## 1. Introduction

Schistosomiasis is a parasitic disease caused by several species of trematodes (platyhelminthes infection or “flukes”) [1]. Among the human parasitic diseases, schistosomiasis ranks second behind malaria in terms of socioeconomic and public health importance in tropical and subtropical areas [2]. Globally, more than 250 million people are affected annually by this disease, in 76 tropical countries, with about 770 million at risk of getting infected and this number is estimated to increase as population growth increases [3]. About 279,000 succumb to the disease annually and a burden of disability is rated at 3.2 million worldwide [3]. In addition to infecting humans, *S. mansoni* also naturally infects nonhuman primates and rodents and these hosts can maintain and transmit the infection in the wild [4].

Schistosomes have a complex life cycle involving parasitic forms in a snail intermediate host, free-living larval forms, and parasitic stages in a definitive host [5]. After penetration and establishment of the parasite, two phases of disease progression are seen; these are the acute and chronic phases [6]. The acute phase occurs between weeks 6 and 12 during which large granulomas form in the liver and intestines. Note that, granulomatous inflammation in schistosomiasis is a defense mechanism elicited by antigens of parasitic eggs mostly trapped in the tissues of the host, mainly in the liver, spleen, and lung [7]. This is a vigorous process coordinated by the immunological response of the host [8]. The development of schistosoma granulomas is mainly as a result of the recruitment of neutrophils and monocytes, accompanied by secretion of TNF-*α*, IL-12, and INF-*γ* [9]. Nevertheless, a typical Th2 response is developed by the host as the infection progresses to being chronic, as a result, more activated macrophages and eosinophils are recruited [10, 11]. The most prominent pathological event that is connected with severe organ injury among schistosoma-infected individuals is granulomatous inflammation [12, 13]. Moreover, during intestinal schistosomiasis, hepatomegaly, splenomegaly, and kidney damage and fibrosis of the bladder are some of the clinical manifestations usually observed [14]. Praziquantel is considered to be safe and effective and is the drug of choice against all forms of schistosomiasis, though reinfection has been reported to occur even after treatment [15].

Human African trypanosomiasis (HAT) also known as sleeping sickness is a neglected tropical disease that affects poor people especially from sub-Saharan Africa and is transmitted through the bites of infected tsetse flies of Glossina species [16]. The disease is caused by infection with the protozoan parasites protozoan *Trypanosoma brucei gambiense* that causes a chronic form of infection in the West and Central Africa and *Trypanosoma brucei rhodesiense* that is associated with the acute form in Eastern and Southern Africa [17].

In trypanosomiasis infection, anemia has been reported as a major laboratory and clinical finding, characterized by a marked reduction in red blood cells, hemoglobin (Hb), and packed cell volume (PCV). Anemia in trypanosomiasis could be due to erythrophagocytosis, hemolytic factor, hemodilution, hematopoietic response, and bone marrow dyserythropoiesis. Anemia as a result of oxidative stress has been linked to trypanosomiasis [18]. Oxidative hemolysis may be associated with high production of free radicals in infected animals [19] and reduced body's endogenous antioxidant reserves.

In addition, reports link trypanosome infection to elevated lipid peroxidation [20] and depleted antioxidants [21]. VSG is frequently linked with T and B lymphocytes and macrophages being activated, resulting in an inflammatory Th1 response, that is linked with the cytokines, TNF-*α*, INF-*γ,* and IL-6, as well as nitric oxide (NO) production [22]. The secretion of proinflammatory cytokine IL-6 during HAT infection has been observed where it induces the production of interferon-*γ* through the early activation of natural killer cells [23]. Three key features exist during the pathogenesis of HAT: anemia, immunosuppression, and tissue lesions [24]. During an infection with HAT, there is evidence that proinflammatory and anti-inflammatory cytokines play a key role in the onset and progression of anemia [25].

Barrier mechanisms of the brain control the movement of cells and other substances between the central and peripheral sections of the brain. These barriers function by severely blocking or preventing this exchange [26]. A wide range of evidence has been documented that there is a probability of the proinflammatory cytokine IFN-*γ* playing an important role in the invasion of trypanosomes in the CNS [27].

Indeed, HAT due to *Trypanosoma brucei rhodesiense* has been observed to provoke multiorgan failure manifested by liver, spleen, and cardiac failure-associated pathologies during the first stage and detrimental neuropathological features in the central nervous system are observed during the second severe stage of the disease [28, 29]. Drug treatment for the severe late stage of HAT relies on intravenous melarsoprol, though very effective the drug is very toxic and painful to administer and is associated with severe post-treatment reactive encephalopathy (PTRE) which develops in 10% of the patients with about half of the fatality cases witnessed [30]. Therefore, the clinical manifestations of HAT are further aggravated with the complications of melarsoprol, resulting in neurological dysfunction generally observed in the severe late stage of HAT infection. Nevertheless, there are new drugs under development for late-stage HAT, such as fexinidazole and nifurtimox-eflornithine combination therapy, which might finally improve the treatment outcome [31, 32].

Several studies have given contradicting information on coinfections. For instance, results from a study by Waknine-Grinberg et al. [33] demonstrate that concomitant *S. mansoni* and *P. berghei* ANKA infection leads to a reduction in cerebral malaria. Another coinfection study reported that *Schistosoma mansoni* infection impairs antimalarial treatment and immune responses of rhesus macaques infected with mosquito-borne *Plasmodium coatneyi* [34]. Despite the natural existence between the parasites that are the major causative agents of HAT and schistosomiasis, the mutual effect of a coinfection by *S. mansoni* and *T. b. rhodesiense* had hitherto not been investigated. There is also little or no information on physiological, biochemical, hematological, and pathological changes due to this coinfection. This study therefore sought to determine *S. mansoni* and *T.b.r* coinfection's ability to reduce or exacerbate inflammation, oxidative stress, and organ pathology.

## 2. Materials and Methods

### 2.1. Parasite Infection Studies

*Schistosoma mansoni* was maintained in the animal house of the Technical University of Kenya by passage through *Biomphalaria pfeifferi* snails. The experimental mice were percutaneously infected with 100 cercariae. Human African trypanosomiasis challenges were performed 9 weeks after *S*. *mansoni*-cercariae infection, which is the period when the mice developed the chronic form of the disease in the coinfection model. Infection with *Trypanosoma brucei rhodesiense-*KETRI 2537 (5.0 × 10^4^ trypanosomes) was performed by intraperitoneal inoculation.

### 2.2. Experimental Design

The study utilized female BALB/c mice aged between 6 and 8 weeks old. The mice were randomly divided into four groups of (*n* = 12) mice; mice were initially infected intraperitoneally with *S. mansoni*, after 9 weeks post-infection again the same group of mice were infected with *T.b. rhodesiense* and then they were followed for a further 3 weeks post-infection. The control group received vehicle only (water). The second group of mice was infected with *T.b. rhodesiense* at a dose of 5 × 10^4^ parasites. The third group was infected with *S. mansoni* (100 cercariae) *and T.b. rhodesiense*. The fourth group of mice was treated with *S. mansoni cercariae* (100 cercariae). All control, infected, and coinfected mice were sacrificed at 21 dpi after *T.b. rhodesiense* infection.

### 2.3. Survival and Parasitemia Determination and Behavioral Assessment

Survival analysis was carried out by monitoring the mice daily for any clinical symptoms and signs and the time and the day at which each mouse succumbed was recorded. The infected group of mice was checked for the presence of parasitemia every two days. In order to assess the parasitemia level, the matching technique developed by Herbert and Lumsden [35] was employed. In this study, a rapid murine coma and behavior scale was employed to determine the neuronal integrity and general health of mice following infection with the two parasites. The experimental mice were assessed according to the parameters related to coordination, exploratory behavior, hygiene-related, reflexes, and self-preservation [36].

### 2.4. Evans Blue Assay

Mice were intravenously injected with 200 *µ*l of 2% Evans blue in 0.95% NaCl (w/v); mice were left to sit for 1 hour and then sacrificed after which brains were removed and then photographed. The brains were weighed and then placed in falcon tubes containing 1 ml of formamide and thereafter incubated for 48 hours at 37°C to extract Evan's blue dye from the tissues. Quantification of the extravasated dye was determined spectrophotometrically at 620 nm.

### 2.5. Hematological, Liver, and Kidney Function Assay and Lipid Profile Determination

At the end of the experiment, blood was collected through cardiac puncture and then placed in EDTA tubes for analysis of hematological parameters using a hematology autoanalyser (Sysmex XS 1000i Hematology Analyzer, WA, USA). For biochemical analysis, blood was collected in Eppendorf tubes. The serum obtained was used to measure the levels of liver and kidney enzyme markers, lipid profile, urea, and uric acid levels by the use of an automatic analyzer (Integra-400 plus analyzer, Roche, Basel, Switzerland).

### 2.6. Cytokines Analysis

Levels of cytokines in serum for IFN-*γ*, TNF-*α,* and IL-10 were evaluated using commercial ELISA kits (Thermo Fisher Scientific Inc., California, USA) in accordance with the manufacturer's protocols. The concentration of serum cytokines was determined using an ELISA plate reader (Thermo Fisher Scientific Inc., Wilmington, MA, USA) at 450 nm absorbance.

### 2.7. Griess Assay

Serum nitric oxide generation was measured by quantifying nitrite which is considered to be the stable end product by employing Griess reagent according to the manufacturer's protocol. In brief, 50 *μ*l of serum was placed into the 96-well plate, followed by the addition of 50 *μ*l sulfanilamide and 50 *μ*l *N*-(1-naphthyl)ethylenediamine dihydrochloride. The absorbance was read at 540 nm and the serum concentrations of nitrite were estimated using a standard nitrite curve.

### 2.8. Reduced Glutathione (GSH) Estimation

The levels of GSH from the brain, liver, kidney, and spleen were measured by employing the method of Griffith [30]. GSH concentration (Sigma-Aldrich Co., St. Louis, MO, USA) was determined by briefly adding 20 *µ*l of both standards and the organ homogenates separately into a 96-well plate in triplicates followed by the addition of Ellman's reagent, the plate was incubated at 37°C for 10 minutes, then the absorbance was read at 405 nm using a microplate reader (Thermo Fisher Scientific Inc., Wilmington, MA, USA).

### 2.9. Malondialdehyde Estimation

To determine lipid peroxidation levels in murine brains, malondialdehyde levels were measured by assays of thiobarbituric acid reactive species (TBARS). Serum samples were mixed with an equal volume of thiobarbituric acid 0.67% and heated at 92−96°C for 30 min. Thiobarbituric acid reactive species production was quantified at 535 nm by using a spectrometer. Results were expressed as malondialdehyde per milligram of protein.

### 2.10. Histopathological Examination: Liver and Brain Histology for Inflammation

The extracted brains and liver were fixed in 4% formalin, the tissue sectioning was performed by a microtome knife following dehydration with ethanol, and then embedded in paraffin followed by hematoxylin and eosin staining. Histopathology lesions were observed microscopically.

### 2.11. Statistical Analysis

Statistical analysis was performed by using the GraphPad prism software package (Version 5.0). One-way ANOVA was performed to compare the infected and coinfected groups with controls. Tukey's post hoc test was employed for internal comparison. Parasitemia was analyzed by unpaired, two-tailed student *t*-test. Survival analysis was performed by the logrank (Mantel-Cox) test. The results were given as a mean ± with significance set at *p* < 0.05.

## 3. Results

### 3.1. Effects of Coinfection on Survival Rate and *T.b.r*-Parasitemia Levels

**Figure 1:**
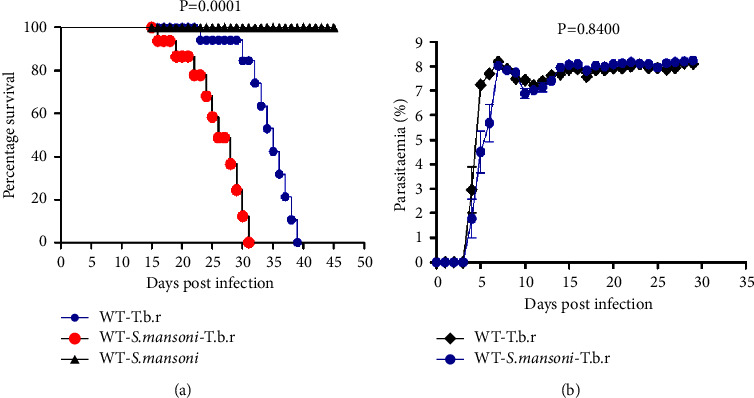
The effects of *T.b.r* and *S. mansoni* coinfection on survival time (a) and *T.b.r*-parasitemia (b) levels. Survival time was analyzed by the logrank (Mantel–Cox) test while parasitemia was analyzed by the unpaired, two-tailed student *t*-test.

This study sought to elucidate the impact of coinfection of mice with *S. mansoni* and *T.b.r* on the outcome of HAT through analysis of the survival time. It was observed that the group of mice coinfected with the two parasites succumbed to the infection within 16 to 31 days post-infection, while mice infected with *T.b.r* alone succumbed to the infection between 23 and 39 days post-infection. Accordingly, the *T.b.r* infected alone group of mice had the median survival time of 35 days post-infection, in comparison to only 26 days post-infection for the coinfected mice. However, the group of mice infected with *S. mansoni* alone registered no mortality, thus the mice in this group were sacrificed after 45 days post-infection due to the development of symptoms of anemia. Overall, mice coinfected with *S. mansoni* and *T.b.r* had statistically shorter survival times relative to either *T.b.r* or *S. mansoni*-infected groups of mice alone at 45 days post-infection (Figure 1(a)). It was surprising to observe that the survival was independent of peripheral *T.b.r*-parasitemia, as an analysis of microscopic parasitemia showed the percentage of *T.b.r*-parasites to be comparable across all the groups (Figure 1(b)).

### 3.2. Effects of *T.b.r* and *S. mansoni* on Rapid Murine Coma and Behavioral Scale, Blood Brain Barrier (BBB), and Evans Blue Extravasation

**Figure 2:**
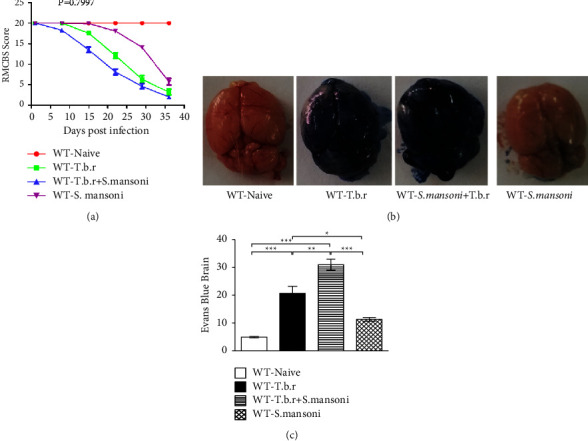
The effects of *T.b.r* and *S. mansoni* on RMCBS, blood-brain barrier, and extravasation of Evans blue dye in the brain. One-way ANOVA was used to compare between various groups followed by the Tukey multiple comparisons post hoc test. Bars represent mean ± SEM. Indicated significance level of ^*∗*^*p* ≤ 0.05, ^*∗∗*^*p* < 0.01, and ^*∗∗∗*^*p* < 0.001.

To demonstrate the neurological injury and health status of the mice, RMCBS analysis was employed in this study. It was observed that at day one, mice from all groups were healthy as depicted by high RMCBS scores. Note that, infection of mice with *T.b.r* alone resulted in a general RMCBS decline from 15 days post-infection, indicating a compromised neurological integrity (Figure 2(a)). In addition, there was a sharp decline in the total RMCBS scores especially in the mice coinfected with *T.b.r* and *S. mansoni*. However, mice infected with *S. mansoni* registered minimal neurological injury relative to a control group of mice. To assess the stability of the BBB, an Evans blue assay was performed. Evans blue binds to serum albumin, which cannot permeate the BBB if it is stable. However, when there is breakage of the BBB, then there is infiltration of the albumin dye into the brain resulting in staining the brain's blue. Mice infected with *T.b.r* (Figure 2(b)) alone exhibited a compromised BBB as depicted by the staining of the brain blue. A similar outcome was observed in the coinfected group of mice with *S. mansoni* and *T.b.r*, suggestive of a compromised BBB. In stark contrast, the brains of mice infected with *S. mansoni* alone and the naïve group of mice remained completely bright. Conspicuously, there was extravasation of the Evans blue dye in the brains of *T.b.r*-infected mice compared to the normal group demonstrated by high colorimetric values (Figure 2(c)). Note that, the amount of Evans blue dye penetrating into the brains of the mice coinfected with the two parasites was significantly increased (*p* < 0.05) relative to the *T.b.r*-infected mice or *S. mansoni* group of mice, demonstrating a compromised BBB.

### 3.3. Effects of *T.b.r* and *S. mansoni* Coinfection on Red Blood Cells, Hemoglobin, and Hematocrit Levels

**Figure 3:**
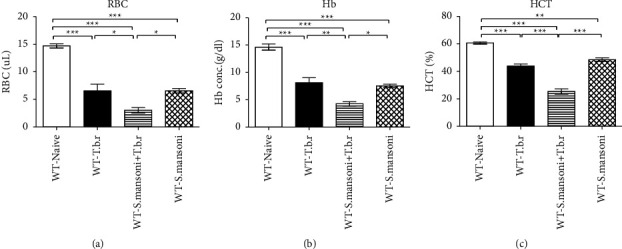
The effects of *T.b.r* and *S. mansoni* coinfection on red blood cells, hemoglobin, and hematocrit levels. One-way ANOVA was used to compare between various groups followed by the Tukey multiple comparisons post hoc test. Bars represent mean ± SEM. Indicated significance level of ^*∗*^*p* ≤ 0.05, ^*∗∗*^*p* < 0.01, and ^*∗∗∗*^*p* < 0.001.

Infection of mice with *T.b.r* or *S. mansoni* alone resulted in a significant (*P* < 0.0001) decrease in the levels of RBC, hemoglobin, and hematocrit (Figures 3(a)–3(c), respectively). Similarly, the coinfected mice with both *S. mansoni* and *T.b.r* registered a further significantly decreased levels of RBC (*P* < 0.0001), hemoglobin (*P* < 0.0001), and hematocrit (*P* < 0.0001).

### 3.4. Effects of *T.b.r* and *S. mansoni* Coinfection on Levels of Red Cell Indices

**Figure 4:**
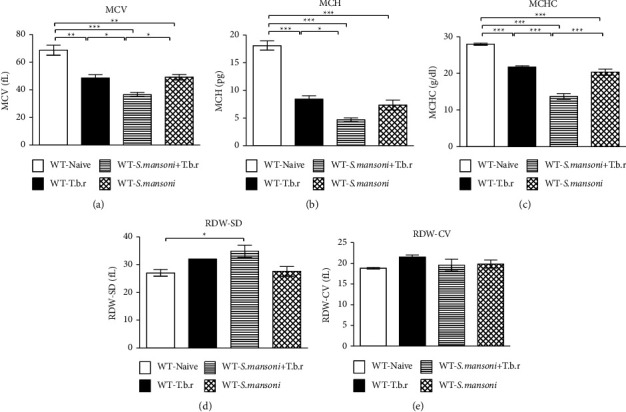
The effects of *T.b.r* and *S. mansoni* coinfection on MCV, MCH, MCHC, RDW-SD, and RDW-CV levels. One-way ANOVA was used to compare between various groups followed by the Tukey multiple comparisons post hoc test. Bars represent mean ± SEM. Indicated significance level of ^*∗*^*p* ≤ 0.05, ^*∗∗*^*p* < 0.01, and ^*∗∗∗*^*p* < 0.001.

There was a significant reduction in the levels of MCV, MCH, and MCHC in mice infected with *T.b.r* or *S. mansoni* alone (Figures 4(a)–4(c)). Consequently, mice coinfected with *S. mansoni* and *T.b.r* resulted in markedly significant low levels of these RBC indices (*P* < 0.0001), indicative of microcytic hypochromic anemia. Exposure of mice to *T.b.r* or *S. mansoni* alone resulted in a significantly lower RDW-SD compared to the levels of control naive mice (Figure 4(d)). However, mice coinfected with *S. mansoni* and *T.b.r* caused marked elevation in the levels of RDW-SD (*P* < 0.0365). There was no significant difference in the levels of RDW-CV (Figure 4(e)) among all groups of mice.

### 3.5. Effects of *T.b.r* and *S. mansoni* Coinfection on White Blood Cells and WBC Differential Count Levels

**Figure 5:**
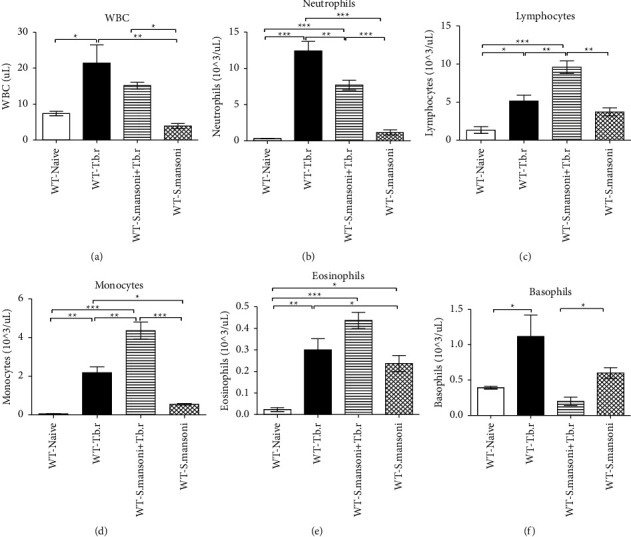
The effects of *T.b.r* and *S. mansoni* coinfection on white blood cells and WBC differential count levels: mice were monitored for neutrophils, lymphocytes, monocytes, eosinophils, and basophils. One-way ANOVA was used to compare between various groups followed by the Tukey multiple comparisons post hoc test. Bars represent mean ± SEM. Indicated significance level of ^*∗*^*p* ≤ 0.05, ^*∗∗*^*p* < 0.01, and ^*∗∗∗*^*p* < 0.001.

Infection of mice with *T.b.r* or *S. mansoni* alone resulted in significantly elevated levels of white blood cells (*P* < 0.0047), WBC differential count of lymphocytes (*P* ≤ 0.0002), neutrophils (*P* < 0.0001), monocytes (*P* < 0.0001), and eosinophils (*P* ≤ 0.0004) (Figures 5(a)–5(e)), respectively, compared to the naïve group. These WBC subtypes were also significantly elevated in mice coinfected with *S. mansoni* and *T.b.r*. Intriguingly, *T.b.r* or *S. mansoni* alone-infected group of mice showed elevated levels of basophils (*P* ≤ 0.0177) compared to the naïve group of mice. However, mice coinfected with *S. mansoni* and *T.b.r* showed a significant (*P* ≤ 0.0177) depletion in the levels of basophil count (Figure 5(f)).

### 3.6. Effects of *T.b.r* and *S. mansoni* Coinfection on Creatinine, Urea, and Uric Acid Levels

**Figure 6:**
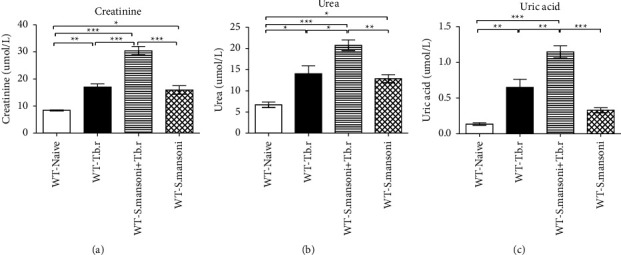
This effects of *T.b.r* and *S. mansoni* coinfection on creatinine (a), urea (b), and uric acid (c) levels. One-way ANOVA was used to compare between various groups followed by the Tukey multiple comparisons post hoc test. Bars represent mean ± SEM. Indicated significance level of ^*∗*^*p* ≤ 0.05, ^*∗∗*^*p* < 0.01, and ^*∗∗∗*^*p* < 0.001.

In this study, creatinine, urea, and uric acid levels were significantly elevated in the *T.b.r* or *S. mansoni* alone-infected group of mice in comparison to the naive group. Consequently, coinfection of mice with *S*. *mansoni* and *T.b.r* resulted in more significantly elevated levels of creatinine, urea, and uric acid (*P* < 0.0001, *P* ≤ 0.0004, and *P* < 0.0001) (Figures 6(a)–6(c), respectively) indicating severe kidney and liver damage.

### 3.7. Effects of *T.b.r* and *S. mansoni* Confection on Liver Damage Markers and Albumin Levels

**Figure 7:**
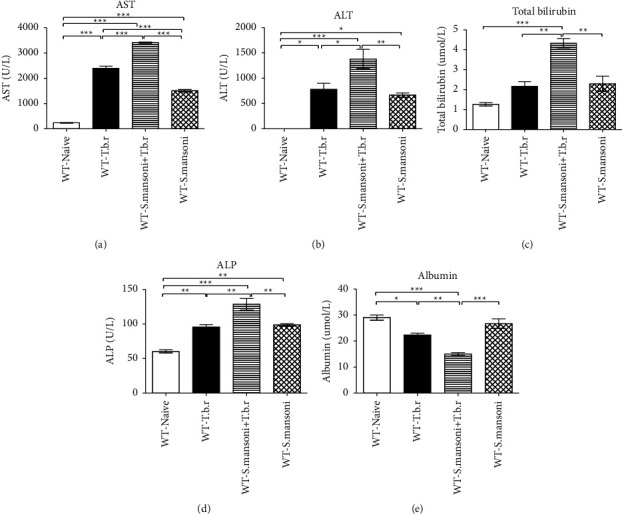
The effects of *T.b.r* and *S. mansoni* coinfection on AST, ALT, total bilirubin, ALP, and albumin. One-way ANOVA was used to compare between various groups followed by the Tukey multiple comparisons post hoc test. Bars represent mean ± SEM. Indicated significance level of ^*∗*^*p* ≤ 0.05, ^*∗∗*^*p* < 0.01, and ^*∗∗∗*^*p* < 0.001.

Infection with *T.b.r* or *S. mansoni* significantly elevated the levels of serum ALT, AST, total bilirubin, alkaline phosphatase, and bilirubin in comparison to naive group mice. In a similar manner, significantly higher levels of ALT and AST were noted among the group of mice coinfected with *T.b.r* and *S. mansoni* (*P* ≤ 0.0181 and *P* <  0.0001; *P* ≤ 0.0008, *P* ≤ 0.0002, and *P* <  0.0001) (Figures 7(a)–7(d), respectively), denoting liver pathology. However, albumin levels decreased significantly in mice infected with *T.b.r* or *S. mansoni* alone in comparison to the naive group (*P* ≤ 0.0001; Figure 7(e)). Similarly, coinfection of mice with *S. mansoni* and *T.b.r* resulted in markedly depleted albumin levels indicative of both liver and kidney damage (Figure 7(e)).

### 3.8. Effects of *T.b.r* and *S. mansoni* Coinfection on Lipid Profile Levels

**Figure 8:**
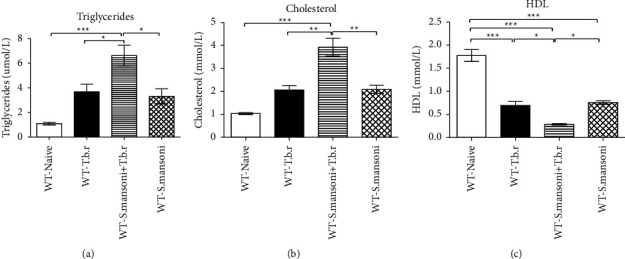
The effects of *T.b.r* and *S. mansoni* coinfection on lipid profile levels of triglycerides, cholesterol, and HDL. One-way ANOVA was used to compare between various groups followed by the Tukey multiple comparisons post hoc test. Bars represent mean ± SEM. Indicated significance level of ^*∗*^*p* ≤ 0.05, ^*∗∗*^*p* < 0.01, and ^*∗∗∗*^*p* < 0.001.

Findings from this study showed that mice infected with *T.b.r* or *S. mansoni* alone registered significant elevation in cholesterol and triglyceride levels in comparison to a naive group of mice (Figures 8(a) and 8(b)). Noticeably, mice coinfected with the two parasites had significantly elevated levels of cholesterol and triglycerides. Meanwhile, *T.b.r* or *S. mansoni* alone-infected mice showed significantly diminished high-density lipoproteins in comparison to the naive group of mice. Coinfection of mice with *S. mansoni* and *T.b.r,* however, resulted in a more significant (*P* < 0.0001; Figure 8(c)) decrease in HDL levels.

### 3.9. Effects of *T.b.r* and *S. mansoni* Coinfection on Proinflammatory and Anti-Inflammatory Cytokine Levels

**Figure 9:**
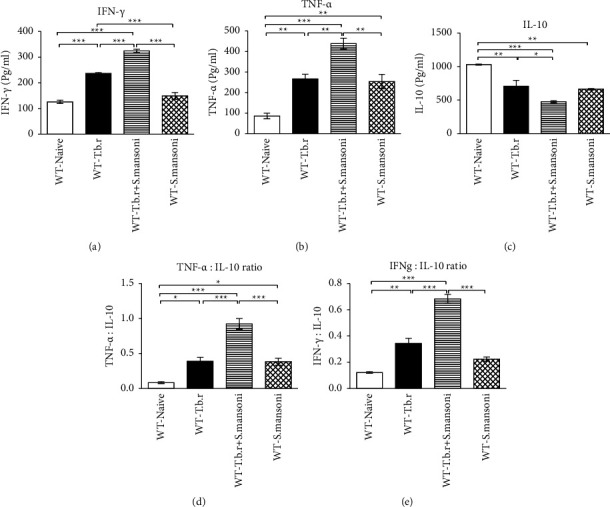
The effects of *T.b.r* and *S. mansoni* coinfection on proinflammatory and anti-inflammatory cytokine levels and ratio. One-way ANOVA was used to compare between various groups followed by the Tukey multiple comparisons post hoc test. Bars represent mean ± SEM. Indicated significance level of ^*∗*^*p* ≤ 0.05, ^*∗∗*^*p* < 0.01, and ^*∗∗∗*^*p* < 0.001.

Serum levels of IFN-*γ* and TNF-*α* were measured from the serum samples to assess the extent of inflammation during the infection process. Results from this study clearly indicate a significant elevation (*P* < 0.0001) of serum IFN-*γ* and TNF-*α* in mice infected with either *T.b.r* or *S. mansoni* alone relative to a naive group of mice (Figures 9(a) and 9(b)). Interestingly, mice coinfected with *S. mansoni* and *T.b.r* showed a markedly pronounced elevation of these proinflammatory cytokines. Furthermore, mice infected with *T.b.r* or *S. mansoni* alone exhibited a significant reduction in serum IL-10 in comparison to the naïve group of mice (*P* ≤ 0.0008; Figure 9(c)). Remarkably coinfection of mice with *S. mansoni* and *T.b.r* resulted in significant (*P* ≤ 0.0008) downregulation of serum IL-10. A further analysis showed that the ratio of the proinflammatory to anti-inflammatory cytokines TNF-*α* : IL-10 (Figure 9(d)) and INF-*γ*- : IL-10 ratio (Figure 9(e)) in mice infected with *T.b.r* or *S. mansoni* alone was significantly elevated (*P* < 0.0001) in comparison to the naive group of mice. Moreover, mice coinfected with *S. mansoni* and *T.b.r* registered a pronounced imbalance between the proinflammatory and anti-inflammatory cytokines.

### 3.10. Effects of *T.b.r* and *S. mansoni* Coinfection on Cellular-Reduced Glutathione Levels

**Figure 10:**
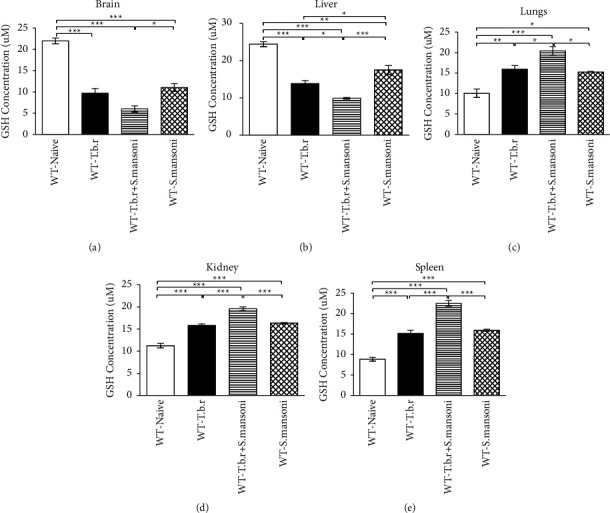
The effects of *T.b.r* and *S. mansoni* coinfection on GSH levels in the brain, liver, lungs, kidney, and spleen. One-way ANOVA was used to compare between various groups followed by the Tukey multiple comparisons post hoc test. Bars represent mean ± SEM. Indicated significance level of ^*∗*^*p* ≤ 0.05, ^*∗∗*^*p* < 0.01, and ^*∗∗∗*^*p* < 0.001.

Infection of mice with *T.b.r* or *S. mansoni* alone resulted in significant (*P* < 0.0001) depletion of GSH levels in the liver and brain (Figures 10(a) and 10(b)) compared to the naïve group of mice. Herein, coinfection of mice with *S. mansoni* and *T.b.r* resulted in a more marked decrease in liver and brain cellular GSH levels, denoting active oxidative stress. Interestingly, lung, kidney, and spleen cellular GSH levels were significantly elevated in mice infected with *T.b.r* or *S. mansoni* alone compared to the levels in the naive group of mice. An elevated level of cellular GSH was equally observed in the lungs, kidney, and spleen from mice infected with both *S. mansoni* and *T.b.r* (Figures 10(c)–10(e)).

### 3.11. Effects of *T.b.r* and *S. mansoni* Coinfection on Malondialdehyde and Nitric Oxide Levels

**Figure 11:**
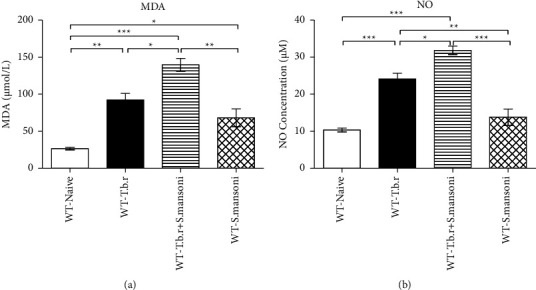
The effects of *T.b.r* and *S. mansoni* coinfection on malondialdehyde and nitric oxide levels. One-way ANOVA was used to compare between various groups followed by the Tukey multiple comparisons post hoc test. Bars represent mean ± SEM. Indicated significance level of ^*∗*^*p* ≤ 0.05, ^*∗∗*^*p* < 0.01, and ^*∗∗∗*^*p* < 0.001.

Mice infected with *T.b.r* or *S. mansoni* showed significantly elevated levels of MDA when compared to the naïve group of mice (*P* ≤ 0.0001). Note that, markedly elevated levels of serum MDA were observed in the group of mice coinfected with *T.b.r* and *S. mansoni* relative to the naïve group (Figure 11(a)), signaling active lipid peroxidation event. On the other hand*, T.b.r* or *S. mansoni* alone-infected group of mice exhibited a significant elevation of NO levels when compared to the naïve group mice. Furthermore, *S. mansoni* and *T.b.r*-infected group of mice portrayed more significantly elevated levels of NO (Figure 11(b)).

### 3.12. Effects of *T.b.r* and *S. mansoni* on Pathology of Mice Liver and Brain Tissues

**Figure 12:**
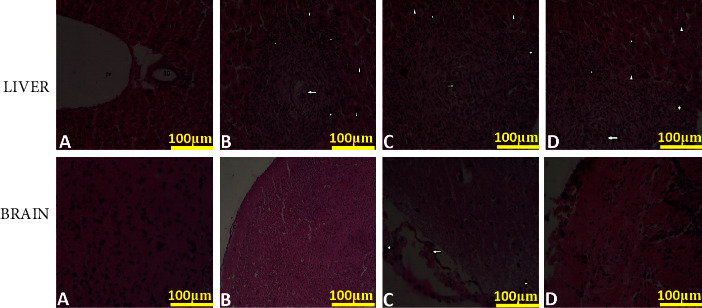
The images show the effects of *T.b.r* and *S. mansoni* infection on liver and brain tissue of mice. Liver and brain tissues from the control group (A), WT-*T.b.r* group (B), WT-*S. mansoni*-*T.b.r* group (C), and WT-*S. mansoni* group (D) were processed for histology with H&E staining. Original magnification ×400 and scale bars = 100 *μ*m.

The liver sections from the naïve control group of mice demonstrated no form of inflammatory lesions or injury (Figure 12(A)) However, mice infected with *T.b.r* or *S. mansoni* alone showed hepatic injury characterized by multifocal granulomas surrounding parasite eggs (arrow) (Figure 12(B)) and multifocal granulomas, marked by connective tissue proliferation (star) and infiltration by lymphocytes, Kupffer cells, and hepatocyte necrosis (arrowhead). Liver sections from mice coinfected with *S. mansoni* and *T.b.r* revealed chronic liver injury characterized by granulomas surrounding parasite eggs (arrow), connective tissue proliferation, and infiltration by lymphocytes (star), Kupffer cells, and hepatocyte necrosis. Histopathological examination of the brain was further performed in this study to determine if there was any inflammation or pathology. Brains from the naive group of mice did not show any sign of injury (Figure 12(B)), whereas brains from mice infected with *T.b.r* or *S. mansoni* alone showed features of brain injury that were characterized by congestion of meningeal blood vessels and focal areas of brain hemorrhages (arrow) and infiltration of meninges with mononuclear cells (star). In addition, mice coinfected with *S. mansoni* and *T.b.r* showed chronic brain injury due to proinflammatory cytokines characterized by focal areas of brain hemorrhages (arrow) and infiltration of meninges with mononuclear cells (star).

## 4. Discussion

From this study, it was established that mice coinfected with the two parasites succumbed to the infection before any of the other groups. However, mice infected with *S. mansoni* alone registered no mortality. Note that, the survival was independent of peripheral parasitemia, as parasitemia was comparable across all the groups.

The rapid murine coma and behavior scale (RMCBS) test was used to determine the impact of the two parasites on the neurological integrity and the general well-being of mice under study [36]. Note that, the most significant drop in neuronal integrity was recorded in the coinfected group. The *T.b.r* infected group presented with low RMCBS scores from 15 days post-infection (dpi) while the *S. mansoni*-infected group did not register a significant change in RMCBS scores. However, a sharp decline in the combined RMCBS scores was recorded in the coinfected group indicating a compromised neuronal integrity and poor health.

The meningoencephalitis or late-stage HAT is characterized by a breach of the blood-brain barrier [26, 37]. In the current study, Evans blue assay was used to assess the stability of the blood-brain barrier. Evans Blue dye cannot permeate the blood-brain barrier if it is stable [38]. However, when there is breakage of the blood-brain barrier, there is an infiltration of the albumin dye into the brain that consequently absorbs the dye turning blue. From the results obtained, it was clear that the coinfected group exhibited a compromised blood-brain barrier as depicted by the brain staining deep blue. A similar outcome was observed in the *T.b.r* infected group suggestive of a compromised blood-brain barrier. The amount of Evans blue dye penetrating the brains of the mice in the coinfected group was significantly high relative to the *T.b.r* infected mice or the *S. mansoni* group of mice, demonstrating a severely compromised blood-brain barrier. Streptococcus pneumoniae and group B streptococci have been reported to cross the blood-brain barrier through transendothelial endocytosis while *Treponema pallidum* and *Borrelia burgdorferi* disrupted tight junctions [39]. Hence, it is not clear how blood-borne parasites cross the blood-brain barrier. In a study by Amrouni et al. [40] , activation of inducible nitric oxide synthase was elevated during the HAT infection process in the central brain compartments particularly in the hypothalamus and thalamus, where regulation of the sleep cycle is located [40]. Possibly, a rise in nitric oxide and proinflammatory cytokines that mediate inflammation could play a role in the breach of the blood-brain barrier in the current study.

Derangement of blood parameters has been shown to have serious consequences in blood-related conditions [24]. Mice infected with *S. mansoni* or *T.b.r* alone had anemia as depicted by the reduced RBC levels, hemoglobin, and HCT. This was replicated in the coinfected group. This outcome is in agreement with a prior coinfection study in which anemia was prevalent among children coinfected with schistosomiasis and malaria [33, 41]. The rise in GSH in the coinfected mice suggests that anemia could be attributed to reactive oxygen species (ROS) generated during infection resulting in oxidative damage on the erythrocyte membranes with subsequent hemolysis [42]. Furthermore, coinfection of the mice with the two parasites specifically induced by microcytic hypochromic anemia is characterized by diminished levels of RBC indices (MCV, MCH, MCHC, and RDW-SD).

The WBC levels were elevated in *T.b.r* and in coinfected groups in this study. This is an expected scenario in most infection processes including those driven by bacteria [43]. This observation may suggest that coinfection of *T.b.r* and *S. mansoni* is immunostimulatory rather than immunosuppressive [44]. In the present study, the *S. mansoni* mice had significantly lower mean levels of total WBC count relative to the control group. This result is in agreement with the results from human studies [45, 46]. The observed low levels of WBC count may be attributed to the amplified intensity of infection and since *S. mansoni* are known to manipulate the host immune response for survival. Therefore, observed low levels of total WBC count due to *S. mansoni* infection may be associated with the immune response to the parasite with concomitant-induced biased proliferation or reduction in specific immune cells regarding the stage of the disease and infection intensity [47].

The levels of monocytes, lymphocytes, and eosinophils were elevated in the coinfected group when compared to the *T.b.r* or *S. mansoni*-infected groups, with basophils levels depleted in the same group. Similarly, neutrophil levels were elevated in the coinfected (*S. mansoni* + *T.b.r*) and *T.b.r* groups. Note that, previous studies have revealed that elevated levels of neutrophils and monocytes activate the production of the proinflammatory cytokines including IFN-*γ*, TNF-*α*, IL-6, and IL-8 leading to inflammation [48]. This may explain why coinfections exacerbate disease conditions associated with inflammation.

Serum levels of urea and creatinine are often used for determining the functional status of the kidneys [49]. Creatinine is a byproduct of creatine phosphate in the muscle while urea is a key product of nitrogenous amino acid and protein catabolism where the end product is secreted by the liver [49]. Estimation of serum urea is useful in the diagnosis of both prerenal condition and acute renal failure [50]. *T.b.r* or *S. mansoni* alone-infected mice had elevated levels of urea and creatinine signaling kidney damage. Remarkably, coinfection of mice with *S. mansoni* and *T.b.r* resulted in a two-fold elevation of urea and creatinine; an indication of a more severe damage to the kidneys.

To assess the extent of kidney and liver injury, uric acid levels were estimated. Uric acid is a waste product released during purine catabolism [51, 52]. In the present study, elevated levels of uric acid were evident in the coinfected group when compared to the *T.b.r* or *S. mansoni*-infected groups. Its accumulation can be due to increased production of purines with the unavailability of uricase enzyme [53]. Uric acid has antioxidant properties when present in body fluids at normal physiological levels. In contrast, at higher concentrations, it becomes proinflammatory [54] and elevated uric acid is implicated in kidney stone formation [51]. A finding from the current study shows more liver damage in the coinfected group when compared to the other groups.

Liver enzyme assays are critical in the diagnosis of the extent of liver damage or injury due to toxic chemicals or diseases. An increase in these markers is a key indicator of liver damage [55]. In this study, there were elevated levels of ALT, ALP, AST, total bilirubin, and albumin in all the infected groups indicating hepatocellular injury. However, these markers were markedly elevated in the coinfected group. Note that, ALT is usually present in the heart, kidney, and muscle, with a higher concentration in the liver than in other body tissues. An increase in ALT signifies liver damage. On the other hand, AST is found in higher concentration in the heart than in the liver, kidney, and skeletal muscles [2].

Bilirubin, a byproduct of hemoglobin is synthesized by the reticuloendothelial system and released as free bilirubin. It then moves to the liver where it is changed to the conjugated form. Levels of direct and total bilirubin were elevated in all the infected groups with heightened elevated levels recorded in the coinfected group. The induction hemolysis of the RBCs led to elevated bilirubin levels suggestive of hepatic diseases. Quick destruction of the red blood cells within the bone marrow yields bilirubin that builds up in the liver leading to inflammation and tissue damage [56].

Serum albumin is synthesized in the liver and has several physiological roles. In this study, albumin levels were very low in the coinfected group than in the other infected groups indicative of liver and kidney damage [57]. Functionally, albumin facilitates the process of coagulation, microvascular permeability, and pH maintenance [58]. In addition, it has antioxidant properties [59].

All the infected groups showed elevation of triglycerides, cholesterol, and a decrease in high-density lipoproteins. However, the coinfected group demonstrated significant elevation of triglycerides, cholesterol, and a decrease in high-density lipoproteins. Late-stage HAT may interfere directly with the regulation of lipid metabolism. It is well documented that HAT is linked to marked alterations in the levels and composition of host lipids and this could be useful in monitoring disease progression [60]. A study by Waema et al. [61] reported high cholesterol levels that were constant with the observed hyperlipidemia in a vervet monkey model infected with *Trypanosoma brucei rhodesiense*. A prior study by Rong et al. [59] observed a substantial depletion in the levels of serum lipid profile among mice that were infected with *S. mansoni*. In the study by Rong et al. [62], reasonably elevated triglyceride levels were detected though LDL and HDL were depleted. In addition, a significant depletion in the mean total cholesterol among subjects infected as seen, could partially account for the reduced mean low-density lipoprotein cholesterol and high-density lipoprotein cholesterol in the same individuals. This could be due to the progressive relationship between HDL-C, total cholesterol, and LDL-C that has been extensively described among normal individuals. Low triglyceride levels among subjects that are infected with *S. mansoni* remain unclear but Felici et al. [63] had proved the capability of *S. mansoni* in synthesizing triacylglycerols and phospholipids from precursors acquired from the host.

Cytokines have an immunomodulatory function in the mammalian system [64]. Proinflammatory cytokines TNF-*α* and IFN-*γ* were significantly elevated in all the infected groups. However, there was a two-fold increase in the levels of these cytokines in the coinfected group. These findings corroborate with the results reported by Lima et al. [65] involving coinfection of *S. mansoni* and *Paracoccidioides brasiliensis* in a mouse model that reflected the reorganization of cells in schistosomiasis as a spontaneous effect of the start of an involute phase of granulomatous. Interleukin-10 was significantly depleted by *T.b.r* or *S. mansoni* infections and further depleted due to coinfection. Increased production of proinflammatory cytokines following infection with either *T.b.r* or S. *mansoni* was evident as demonstrated by the ratio of proinflammatory to anti-inflammatory cytokines. Coinfection created a greater imbalance between the proinflammatory and anti-inflammatory cytokines. Since Th2 cytokines such as IL-4, IL-5, and IL-13 are implicated during schistosomiasis, it is worthy to evaluate such interleukins during the coinfection with human African trypanosomiasis.

Levels of reduced glutathione (GSH) are often used to determine the level of oxidative stress in tissues. Coinfection with *T.b.r* and *S. mansoni* resulted in elevated GSH levels in the kidney, lungs, and spleen. In addition, there was depletion of GSH in the brain and liver, which is an indication of severe oxidative stress following coinfection.

To further validate GSH findings on oxidative stress, malondialdehyde (MDA) and nitric oxide (NO) estimation assays were performed. Elevated levels of MDA and nitric oxide were recorded in all the infected groups. However, there was a more significant elevation of MDA and NO in the coinfected group, signaling severe oxidative damage. Elevated levels of NO are usually an indicator of underlying inflammation [66]. The heightened destruction of erythrocytes and lymphocytes often leads to the release of MDA as well as reduced GSH [67]. The commonly used oxidative stress biomarker is MDA [68]. MDA production is a relevant indicator of lipid peroxidation *in situ* [69]. Nitric oxide also functions as an endothelium and endogenous relaxing factor and as a free radical. NOS enzyme produces NO which can drive the formation of free radicals in various diseases [70]. Nitric oxide can lead to the formation of peroxynitrite a very reactive molecule that damages DNA [71].

A standard histopathology analysis of the brain, liver, and spleen sections was performed in the current study. Liver sections from mice coinfected with the two parasites had an abnormal hepatocyte distribution characterized by granulomas, connective tissue proliferation, and infiltration by lymphocytes, Kupffer cells, and hepatocyte necrosis [72]. The findings validate the earlier outcomes in the current study that gave an indication of liver damage as depicted by the elevated liver biomarkers. Brain sections from mice infected with *T.b.r* or *S. mansoni* alone had congestion of meningeal blood vessels. In addition, brain sections from mice coinfected with *T.b.r* and *S. mansoni* exhibited meningitis characterized by focal areas of brain hemorrhages and infiltration of meninges with mononuclear cells.

## 5. Conclusions

The present study provides compelling evidence that coinfection of mice with *S. mansoni* and *T.b.r* results in adverse effects linked to alteration of biochemical and physiological functions, exacerbated neurobehavioral deficits, organ pathology, and compromised immune response with broad and significant implications in disease progression, diagnosis, and treatment. Data from this study have also demonstrated that coinfection (*S. mansoni* and *T.b.r*) results in more severe physiological and biochemical changes. This phenomenon requires further scrutiny to unravel implications in the treatment and management of *S. mansoni* and *T.b.r*.

## Data Availability

The data used to support the findings of this study are available from the corresponding author upon request.
